# Adjuvant radiotherapy and chemotherapy in breast cancer: 30 year follow-up of survival

**DOI:** 10.1186/1471-2407-10-398

**Published:** 2010-07-30

**Authors:** Colin S McArdle, Donald C McMillan, Nicola Greenlaw, David S Morrison

**Affiliations:** 1 University Department of Surgery, Faculty of Medicine, University of Glasgow, Royal Infirmary, Glasgow, UK, G31 2ER; 2 West of Scotland Cancer Surveillance Unit, Section of Public Health and Health Policy, Faculty of Medicine, University of Glasgow, Glasgow, UK, G12 8RZ

## Abstract

**Background:**

The long term outcome (more than 15 years) of adjuvant treatment in patients with primary operable breast cancer has rarely been examined.

**Methods:**

A randomised clinical trial of radiotherapy, chemotherapy (28 day cycles of cyclophosphamide, methotrexate and 5-fluorouracil) or both on women with primary operable breast cancer (n = 322) was followed-up for a median of 27 years.

**Results:**

260 (81%) patients died, 204 (78%) from breast cancer. Cancer specific survival (SE) at 10 years, 20 years and 30 years was 41 (3)%, 34 (3)% and 33 (3)% respectively. Presence of more than 3 involved lymph nodes increased cancer-specific mortality (HR 1.88, 95% CI 1.34-2.63) after adjustment for age, socio-economic deprivation and adjuvant treatment. Both age (HR 1.63, 95% CI 1.19-2.22) and involved lymph nodes (HR 1.59, 95% CI 1.17-2.14) were significant predictors of all-cause mortality after adjustment for other factors. There was no significant difference in all-cause or cancer-specific survival between patients in each of the 3 treatment arms.

**Conclusions:**

The present study highlights the long term impact of node positive disease but does not indicate that any regimen was associated with significantly better long-term survival.

## Background

Breast cancer is the commonest malignancy in females and is a major cause of morbidity and mortality in the Western World[1]. Prior to the introduction of chemotherapy the mainstay of treatment was based on surgery and radiotherapy. A series of clinical trials started in the mid 1970 s, with approximately 5 years' follow-up, established the role of chemotherapy as an adjuvant treatment for primary operable breast cancer[2-5].

In contrast, the long term outcome of such adjuvant treatment in these patients is less clear. Recently, Bonadonna and co-workers[6] reported that, after follow-up of 30 years, adjuvant cyclophosphamide, methotrexate, and fluorouracil (CMF) was associated with a persistent reduction in overall mortality of approximately 20% compared with surgery alone. However, to our knowledge, there have been no reports that compare long term survival in patients receiving CMF with two of the commonest alternative regimens for primary operable breast cancer, CMF combined with radiotherapy and radiotherapy alone[3].

The aim the present study was therefore to establish long term outcome in women receiving adjuvant radiotherapy and chemotherapy as part of a randomised trial.

## Methods

The study design has been previously described[3]. Briefly, between June 1976 and December 1982 women aged 70 years or less admitted to three teaching hospitals, with operable breast cancer and with no evidence of metastatic disease, were entered into the study. Women with histological involvement of the axillary nodes were randomized to receive either: conventional postoperative radiotherapy, chemotherapy alone or radiotherapy followed by chemotherapy. The chemotherapy regimen was based on that described by Bonadonna - consecutive 28 day cycles of cyclophosphamide, methotrexate and 5-fluorouracil[2].

Information on date and cause of death was obtained through Scottish Cancer Registry patient-based linkage with the General Register Office for Scotland (GRO(S)) death records. Deaths up to the end of 30^th ^September 2007 have been included in the analysis, providing a median length of follow-up of 27 years (minimum 25 years, maximum 31 years). Cancer-specific survival was calculated using all deaths in which the principal cause of death was recorded as breast cancer (International Classification of Diseases, Revision 9, 174 and 199; ICD-10 C50). Socio-economic deprivation was inferred for each patient using the DEPCAT score[7] associated with their postcode of residence. The DEPCAT is a validated 7-category score that ranks all postcode areas from 1 (most affluent) to 7 (most deprived) using four Census variables shown best to correlate with health outcomes: car ownership; overcrowding; proportion of population in occupational Social Classes IV and V; and male unemployment. We further grouped DEPCATs into 3 conventional classes: DEPCATs 1 and 2 (affluent); 3 to 5 (intermediate); and 6 and 7 (deprived).

### Statistics

Cumulative survival after mastectomy was estimated using the Kaplan-Meier method and the logrank used to test for independence between treatment groups. The grouping of variables was carried out using standard thresholds. Life tables showing cancer-specific survival and associated standard errors were calculated using SPSS version 15 software. Univariate and multivariate survival analysis and calculation of hazard ratios (HRs) were carried out using Cox's proportional-hazards model. Analysis was carried out using STATA version 10 statistical software. Multivariate analyses were stratified by oestrogen receptor status in order to satisfy the proportionality assumption, which was tested using the calculated Schoenfeld residuals and the scaled Schoenfeld residuals[8]. These residuals are used to test the null hypothesis that the log hazard-ratio function is constant over time, that is, that the ratio of hazards between different groups (for example, between patients aged 50 or less and over-50) is constant over time.

## Results

Three hundred and twenty-two patients were included in the study. One hundred and three patients were allocated to receive radiotherapy alone, 111 sequential radiotherapy and chemotherapy and 108 chemotherapy alone. The incidence of unfavourable prognostic features, such as age, deprivation, tumour size, involved lymph nodes, and oestrogen receptor status, was similar between the groups.

**Figure 1:**
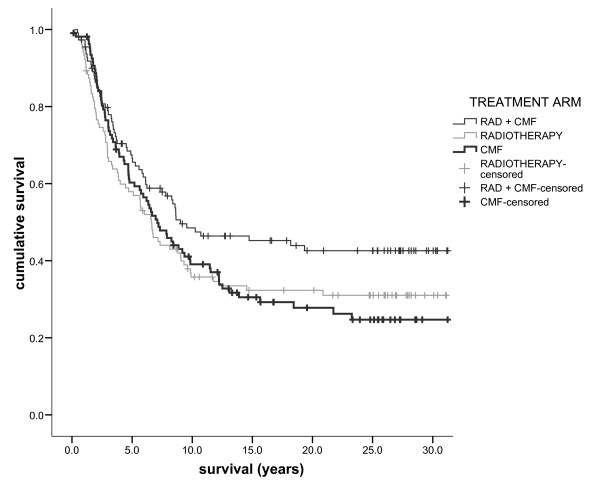
**The relationship between treatment (radiotherapy and chemotherapy/radiotherapy/chemotherapy from top to bottom) and cancer specific survival in patients with primary operable breast cancer with 30 year follow-up**.

After 30 years' follow-up 260 (81%) died; of these 204 (78%) died of breast cancer. Cancer specific survival (SE) at 10 years, 20 years and 30 years was 41 (3)%, 34 (3)% and 33 (3)% respectively. All-cause survival (SE) at 10 years, 20 years and 30 years was 37 (3)%, 23 (2)% and 19 (2)% respectively. There was a trend towards improved cancer-specific survival in the sequential radiotherapy and chemotherapy arm but no overall difference in either breast cancer-specific (Figure 1, logrank p = 0.090) or all-cause survival (p = 0.154) between groups.

The relationships between clinicopathological features and treatment and both cancer-specific and overall survival at 30 years are shown in Table 1. Age over 50 years, and more than 3 involved lymph nodes, were associated with significantly poorer cancer-specific and all-cause survival. Formally defined post-menopausal status was associated with poorer overall survival but not with cancer-specific survival.

**Table 1:** 30-year cancer specific and overall cumulative survival for patients undergoing adjuvant radiotherapy and chemotherapy with primary operable breast cancer, n = 322

	Cancer-specific survival	p-value	Overall survival	p-value
Age				
≤50 years	40 (5)	0.023	31 (2)	0.001
>50 years	28 (3)		12 (2)	
Socio-economic circumstances				
Affluent (DEPCATs 1-2)	41 (6)		24 (5)	
Intermediate (DEPCATs 3-5)	33 (4)	0.657	19 (3)	0.784
Deprived (DEPCATs 6-7)	27 (5)		14 (4)	
Menopausal status				
Pre	39 (4)	0.071	30 (4)	0.002
Post	29 (4)		12 (2)	
Involved lymph node				
≤3	38 (4)	<0.001	21 (3)	<0.001
>3	23 (4)		14 (3)	
Hormonal-receptor status				
ER+	33 (5)	0.052	17 (4)	0.055
ER-	31 (5)		17 (4)	

The relationships between clinicopathological features, treatment and both cancer-specific and overall survival were explored by multivariate proportional hazards analyses and results shown in Table 2. Increasing age (p = 0.018) and involved numbers of lymph nodes (p < 0.001) were associated with poorer cancer specific survival in univariate analysis. On multivariate analysis, shown in Table 2, only involved lymph nodes (HR 1.88, 95% CI 1.34-2.63) remained significant determinants of cancer-specific survival. Both age (HR 1.63, 95% CI 1.19-2.22) and involved lymph nodes (HR 1.59, 95% CI 1.17-2.14) were significant predictors of all-cause mortality after adjustment for other factors. There was no significant difference in all-cause or cancer-specific survival between patients in each of the 3 treatment arms. There were no significant interactions between survival from each of the 3 treatment modalities and age, deprivation or number of involved lymph nodes.

**Table 2:** Multivariate hazards of breast cancer-specific and all cause mortality at 30 years for 322 patients undergoing adjuvant radiotherapy and chemotherapy with primary operable breast cancer, stratified by oestrogen receptor status.

		Cancer-specific	survival	Overall	survival
	Patients (n = 322)	Hazard ratio (95% CI)	*P*-value	Hazard ratio (95% CI)	*P*-value
Age (>50 years)		1.34 (0.95-1.89)	0.097	1.63 (1.19-2.22)	0.002
					
Deprivation (DEPCAT)			0.226		0.322
1-2		1.00		1.00	
3-5		1.04 (0.68-1.60)	0.845	0.94 (0.65-1.36)	0.740
6-7		1.39 (0.90-2.14)	0.144	1.21 (0.82-1.77)	0.332
Involved lymph nodes					
1,2 or 3		1.00		1.00	
≥4		1.88 (1.34-2.63)	<0.001	1.59 (1.17-2.14)	0.003
					
Treatment			0.220		0.309
Radiotherapy		1.24 (0.81-1.90)	0.324	1.02 (0.70-1.48)	0.921
Radiotherapy + CMF		1.00	1.00	1.00	
CMF		1.43 (0.96-2.13)	0.082	1.28 (0.90-1.81)	0.169

CMF, cyclophosphamide, methotrexate and 5-fluorouracil.

## Discussion

In the present study there was an approximate 80% reduction in 30 year cancer-specific survival in those patients with 4 or more involved lymph nodes. In contrast, there was no significant impact of treatment modality on long term cancer survival. Therefore, the present study confirms the long term impact of node positive disease but does not indicate that any regimen was associated with significantly better long-term survival.

The results of the present study are therefore consistent with our previous report of the 5 year results of this randomised trial comparing adjuvant radiotherapy, radiotherapy and chemotherapy and chemotherapy alone in women with primary operable breast cancer[3]. The main findings then were that the combination of radiotherapy and chemotherapy was associated with reduced recurrence and a trend towards increased cancer-specific survival; and that disease-related survival was mainly dependent on the number of positive nodes. The present results are also consistent with other reports which indicate that while radiotherapy reduces the risk of loco-regional recurrences[9,10], it has no effect on mortality at 20 years (although Rutqvist also reported that chemotherapy was associated with longer recurrence-free survival among premenopausal women only)[10]. A meta-analysis suggests that the modest benefits of radiotherapy on breast cancer specific mortality are offset by increased mortality from other causes[11]. While Fisher and others' trial[12] - which began in the same year as the present study - found that the addition of radiotherapy to segmental mastectomy conferred a significant survival advantage, it may be that longer-term follow-up would also have shown increased mortality from others causes. It seems unlikely that differences in surgical interventions between treatment arms - a choice of breast conserving surgery of mastectomy - would have influenced long-term survival[13-15]. A systematic review of chemotherapy trials for breast cancer found that adjuvant polychemotherapy reduced mortality in postmenopausal women by 2% and 6% in node negative and node positive women, respectively[16]. However, the observed effect may have been be due to the additional benefits of tamoxifen on long-term survival[17] and the drug was not available in the United Kingdom at the start of the present study.

The present study has a number of limitations, principally that we did not have data on tumour grade with which we might have been able to calculate the Nottingham Prognostic Index[18] nor did we have more detailed data on doses and compliance with each treatment modality. Death records may be susceptible to coding errors and misclassification of the cause of death might have either increased or decreased the true cause-specific mortality. The lack of statistically significant differences in survival between treatment arms may be due to insufficient numbers of patients; it may reflect a true absence of effect; or unidentified confounding factors may have obscured a true difference. Nevertheless, the major strength of the present study is the duration of follow up, which, with the exception of the study by Bonadonna[2], is to our knowledge the longest reported.

## Conclusions

Although there have been advances in the surgical, radiological and chemotherapeutic treatment of primary operable breast cancer over the past 30 years the present results highlight the long lasting detrimental effect of node positive disease. Our analysis describes the impact of the first adjuvant regimens but does not indicate that any regimen was associated with significantly better long-term survival.

## Authors' contributions

CSM designed the study and gathered original clinical data; DSM obtained and linked deaths records; DCM, DSM and NG designed this follow-up study, conducted statistical analyses, and drafted the manuscript; all authors saw and approved the final version of the manuscript.

## Competing interests

The authors declare that they have no competing interests.

## Pre-publication history

The pre-publication history for this paper can be accessed here:

http://www.biomedcentral.com/1471-2407/10/398/prepub
